# Adipocytokines in Thyroid Dysfunction

**DOI:** 10.1155/2013/646271

**Published:** 2013-06-23

**Authors:** Berna İmge Aydogan, Mustafa Sahin

**Affiliations:** Department of Endocrinology and Metabolic Diseases, Ankara University Faculty of Medicine, İbni Sina Hastanesi Ek Bina M-blok Kat:1, Sıhhiye, 06100 Ankara, Turkey

## Abstract

Adipocytokines are important mediators of interorgan crosstalk in metabolic regulation. Thyroid diseases have effects on metabolism and inflammation. The mechanism of these effects is not clear. Recently, there are several reports suggesting this interrelation between adipocytokines and thyroid dysfunction. In this review, we summarize this relation according to the literature.

## 1. Introduction 

Adipose tissue is an active endocrine organ. Recently many adipocytokines are discovered to have role in regulation of metabolism and body composition. Adipocytokines have autocrine, paracrine, and endocrine functions on several organs. They seem to regulate thermogenesis, immunity, feeding, and neuroendocrine functions. There are good and bad adipokines for health. Adipokines have a central role in subclinical inflammation of adipose tissue, and obese adipose tissue secretes proinflammatory adipokines such as leptin, visfatin, and resistin [1, 2]. 

Nutrition also influence the adipokine effect on inflammation [3]. Their role in obesity, metabolic syndrome, diabetes, and cardiovascular disease is well established [4]. Leptin is found to be immune modulator [5]. Adipokines are linked to autoimmune diseases [6]. Adipokines like adiponectin have role in anticarcinogenesis, and they can be used as prognostic factors. Also they can be used in cancer therapy [7].

Thyroid hormones are also crucial for the regulation of total energy consumption and body composition besides their roles in normal growth, development, and reproduction. A positive correlation between serum thyroid stimulating hormone (TSH) levels and body mass index (BMI) is suggested as thyroid dysfunction is associated with weight changes [8, 9]. Serum TSH and free triiodothyronine levels are found to be higher in obese patients [10]. High-energy intake results in an increase of plasma T3 levels, and starvation causes a decrease in plasma T3 (triiodothyronine) levels [11]. Also negative associations between FT4 and body weight in euthyroid subjects were reported [12]. 

Hyperthyroidism and hypothyroidism are also both associated with insulin resistance [13]. The exact pathogenesis of these findings is obscure, but adaptive responses may have role. Positive relations between BMI and triiodothyronine (T3) in both genders were suggested previously [14]. Recently, Roef et al. also showed positive associations between T3 and insulin resistance in euthyroid subjects [15]. 

Although there are conflicting data about the associations between the thyroid hormone and TSH levels with the body composition, it is clear that arcuate nucleus of hypothalamus has the major role in regulation of appetite. Coppola et al. showed that NPY/AgRP neurons are exposed to T3, and T3 can activate these cells and stimulate food intake [16]. On the other side, adipose tissue is confirmed to be a gland that secretes several molecules with multiple metabolic functions. This review suggests a complex interaction between thyroid hormones and adipocytokines. 

## 2. Leptin

Leptin is an adipocyte-derived, 167-amino acid anorexigenic hormone which is a product of obese (ob) gene located on chromosome seven. Major site of leptin production is white adipose tissue, but it can be produced by other tissues like placenta, ovary, mammary epithelial cells, hypophysis, stomach, and liver as well [17]. Leptin receptor (Ob-RL) is a member of the class 1 family of cytokine receptors which is coded by *db *gene and expressed in liver and other peripheral organs besides the predominantly hypothalamic expression [18, 19]. It is well known that leptin is involved in response to fasting. Genetic deficiency of leptin or its receptor cause obesity and obesity-associated diabetes mellitus [20]. The mechanism of leptin's effects on appetite and energy consumption is not only due to central neural regulation. Orexigenic peptides are downregulated and anorexigenic peptides are upregulated by leptin [21]. Additionally, leptin increases thyroid hormone levels [22]. 

Leptin has both acute and well-known long-term effects on metabolism [23]. As it affects thyroid metabolism by indirect effects, it may also affect thyroid axis in acute manner. Leptin administration reverses the fasting induced suppression of hypothalamus-pituitary-thyroid axis at the central level by upregulating TRH expression in the hypothalamus [24]. Ortiga-Carvalho et al. showed that leptin has a stimulatory effect on release of TSH in vivo [25]. Also a direct stimulatory effect of leptin on thyroxine (T4) released from the thyroid gland is suggested [26]. Leptin regulates central and peripheral iodothyronine deiodinase activity and conversion of T4 to T3 [27]. Leptin administration has been shown to prevent the fasting induced changes in serum T3 and T4 concentrations. Araujo et al. demonstrated that leptin administration restores deiodinase 1 activity in rats [28]. Boelen et al. reported that fasting increased liver D3 mRNA in mice and leptin administration restores fasting induced increased hepatic D3 expression independently of serum thyroid hormone concentrations [29]. Leptin also increases D2 activity centrally and leads to an increase of T3 [30]. So, leptin may have role in the pathogenesis of euthyroid sick syndrome. 

Besides TSH stimulates leptin secretion by a direct effect on adipocytes, probably via TSH-receptors on the surface of adipocytes. Positive association between leptin and TSH can be caused by this direct effect of TSH on leptin secretion by adipocytes [31]. 

Serum thyroid hormones also seem to affect the leptin levels. In vivo and in vitro rat studies established that increased serum T3 leads to a deprivation in leptin mRNA expression at white adipose tissue and serum leptin levels [32]. 

Roef et al. demonstrated associations of free thyroid hormone levels with body composition and metabolic parameters in a population of healthy euthyroid men [15]. In their study, thyroid hormone levels were strongly associated with adiposity indices, and negative associations were observed with parameters of muscularity. They hypothesized that effects of body fat on thyroid hormone levels can be explained through thyroxine-binding globulin but after adjustment for TBG; body composition and metabolic parameters were still associated. A positive association between leptin or fat mass and the ratio of FT3 to FT4 was observed. Serum TSH levels were also associated with leptin levels but not with body composition. Both thyroid hormones and leptin affect each other and may regulate body composition and metabolism by complex mechanisms. 

It is also suggested that leptin and its receptors have a role in the pathogenesis of thyroid cancer. Leptin regulates multiple signaling pathways in different cancer types. JAK2/STAT3 receptor is a thyrosine kinase receptor, and it can activate thyrosine kinase JAK2 [33]. Leptin stimulates the phosphatidylinositide 3-kinases (PI3 K) pathway and activation of AKT causes the expression of proteins which leads to cell proliferation and apoptosis inhibition [34]. 

In a recent study, immunohistochemical staining with leptin receptor antibody on PTC tumor samples demonstrated that expression of Ob-R protein was detected in 80% of samples. Leptin receptor expression was associated with older age, greater tumor size, extrathyroidal extension, lymph node metastasis, and advanced tumor stage, so in summary aggressive behavior of PTC and poor disease survival were observed [35]. 

Akinci et al. [36] showed that PTC patients had significantly higher levels of leptin when compared to healthy subjects. In their study total thyroidectomy caused a decrease of serum leptin levels in all BMI subgroups. So leptin also may have role in pathogenesis and prognosis of thyroid cancers. 

## 3. Ghrelin

Ghrelin is an acylated 28-amino acid peptide hormone and a ligand of growth hormone secretagogue receptor (GSH-R) [37]. It was firstly isolated from rat stomach. Gastric mucosa neuroendocrine cells are the major site of secretion but also hypothalamus, hypophysis, pancreas, kidney, intestines, placenta, and other tissues can produce ghrelin [38]. It is known that ghrelin regulates energy balance, appetitive behaviour, and thermogenesis and stimulates GH release at the arcuate nucleus of hypothalamus where NPY- and AgRP are also expressed [39–41]. It is suggested that ghrelin's regulatory effects on appetite are due to NPY activation [42]. In fasting and other catabolic conditions the level of ghrelin increases and also intravenous administration of ghrelin stimulates appetite [43, 44]. Ghrelin is a physiological antagonist of leptin; it leads adiposity and obesity, so in obese patients ghrelin levels decrease as well. 

It is demonstrated that GHS-R mRNA and ghrelin are both present in thyroid gland [45]. Some studies showed that ghrelin levels decrease in hyperthyroidism as compared to euthyroid state and return to normal ranges after treatment of hyperthyroidism [46]. Subclinical hyperthyroidism was not associated with changes in ghrelin levels [47]. In a rat study, hyperthyroidism was associated with decreased serum ghrelin levels and increased gastric mucosa ghrelin immunoreactivity [48]. Mechanism of this ghrelin reduction is not clear and it is suggested that hyperinsulinemia in hyperthyroidism or excess thyroid hormone can both have a role. 

Hypothyroidism was also reported to be associated with increased levels of ghrelin. In a recent study ghrelin had a stimulating effect on fT4 secretion in vivo [49]. On the other side some investigators reported normal levels of serum ghrelin in hypothyroidism [50]. Altinova et al. demonstrated that ghrelin levels were lower in Hashimoto thyroiditis patients compared with the healthy subjects [51]. Thyroid peroxidase antibody titers were associated with lower ghrelin concentrations. It is suggested that the coincidence of atrophic gastritis with autoimmune thyroid disease can be the reason of decreased ghrelin production from gastric cells in those patients [52]. 

A study of total thyroidectomized patients for thyroid cancer, recombinant TSH administration caused suppression of serum ghrelin levels independently, and investigators suggested that TSH may have a stimulatory effect on ghrelin secretion at gastric mucosa [53]. But none of the studies exactly measure quantitative food intake ant its relation with ghrelin. 

The data about expression of ghrelin in normal human thyroid gland is conflicting, but a relationship between thyroid cancers and ghrelin has already been suggested by some authors. In human thyroid gland C cells seem to be responsible for ghrelin production. Raghay et al. [54] demonstrated that there was a ghrelin immunoreactivity in all types of differentiated thyroid tumors, but another study did not support this finding as ghrelin levels in thyroid tissue were lower in papillary thyroid carcinoma compared with normal thyroid gland [55]. It is suggested that the association between ghrelin tissue concentrations and thyroid cancer can be explained with in vitro antiproliferative effects of ghrelin [56]. However, Morillo-Bernal et al. recently demonstrated that ghrelin potentiates TSH-induced cell proliferation and has stimulatory effect in expression of thyroglobulin, thyroid peroxidase, and sodium iodine transporter genes at rat thyrocytes [57]. 

## 4. Obestatin 

Obestatin is another newly described adipocytokine which is a ghrelin-associated peptide. Major site of its expression is stomach. It is believed that obestatin does not have any activity by itself [58]. However, its effects on body composition are not still clear. The association between thyroid diseases and obestatin levels was rarely studied. Kosowicz and collegues demonstrated that hypothyroidism is associated with high and hyperthyroidism is associated with low levels of obestatin [59]. Gurgul et al. found a positive association between TSH levels, ghrelin, and obestatin in hypothyroidism and suggested that obestatin may be a modulatory molecule [60]. 

## 5. Visfatin 

Visfatin is a visceral fat-derived adipocytokine also called as pre-B cell colony-enhancing factor. It is a large protein and its gene is located on chromosome 7. Pre-B cell colony-enhancing factor was firstly demonstrated as a product of peripheral blood leucocytes [61]. Further the molecule was reported to be expressed in adipose tissue [62]. 

Visfatin is an insulin mimetic molecule and has an important role in adipocyte differentiation [63]. A positive correlation between visfatin levels and obesity was demonstrated previously. Visfatin excess in obesity is thought to be a result of adipose tissue macrophage activation [64]. However, it is now clear that adipose tissue macrophages, hepatocytes, and skeletal muscles can also express visfatin [65–67]. 

As visfatin is supposed to have proinflammatory effects and a rise in serum levels was observed in autoimmune diseases [68], the changes of visfatin levels in thyroid disorders are also attributable to immunological mechanisms. It is suggested that visfatin can activate IL-6, and also IL-6 can stimulate visfatin gene expression [69]. 

In Graves disease patients, visfatin levels found to be elevated and positively correlated with serum T3 and FT4 [70]. On the other side MacLaren et al. demonstrated that T3 can downregulate the visfatin mRNA expression in adipocytes [71]. Han et al. reported that both hyper- and hypothyroidism were associated with higher visfatin levels compared to euthyroid subjects, and they underlined that visceral fat accumulation is an indicator of insulin resistance, and plasma visfatin levels may be determined by increased visfatin mRNA expression in visceral fat [72]. Another hypothesis is the protective visfatin expression from cardiovascular system in thyroid hyper/hypofunction [73]. 

Ozkaya et al. reported that low-visfatin levels were found in hyperthyroid patients compared with the hypothyroid and control groups [74]. Their study showed that visfatin levels in hypothyroidism decreased significantly following treatment and increased significantly after antithyroid therapy in hyperthyroidism. Caixàs et al. found that normalization of thyroid function caused an increase of plasma visfatin levels in hypo- and hyperthyroidism [75]. Also basal serum visfatin levels were higher when compared to control in both hyper- and hypothyroidism. Their findings were not supporting the previous reports; they found that visfatin level was not related to inflammatory parameters and insulin resistance. 

## 6. Adiponectin

Adiponectin is a 244-amino acid peptide and it is secreted from the adipose tissue [76]. It acts through two different receptors; adiponectin receptor 1 (AdipoR1) is expressed predominantly in muscle, and adiponectin receptor 2 (AdipoR2) is expressed predominantly in liver [77]. 

In obesity and insulin resistance, serum adiponectin levels decrease [78]. After weight loss or treatment of insulin sensitivity, adiponectin levels increase as well [79]. It is also suggested that low-adiponectin levels are correlated with coronary artery disease [80]. 

Because of their similar effects on metabolism, thyroid hormones and adiponectin are believed to be associated with each other independently from weight status. It was reported that adiponectin levels were higher in hyperthyroidism compared to hypothyroidism or euthyroidism, respectively, [81, 82]. Conversely Santini et al. and Iglesias et al. reported that adiponectin levels were not significantly different in hyperthyroidism when compared to control groups [83, 84]. Altinova et al. could not demonstrate a relationship between thyroid status and adiponectin levels [85]. It is also suggested that thyroid hormone levels are associated with adiponectin levels in healthy subjects [86]. 

Adiponectin is supposed to have antiangiogenic and anticancer activity. Adiponectin levels have been found lower than normal in several cancer types [87–89]. Recently, Mitsiades et al. showed that adiponectin concentrations were significantly lower in thyroid carcinoma patients than control group [90]. They demonstrated AdipoR1 and AdipoR2 expression in thyroid carcinoma cell lines. Recombinant adiponectin treatment did not influence cell proliferation or survival in vitro. Their results were attributable to the effect of insulin resistance on thyroid carcinoma development. 

## 7. Resistin

Resistin is a 114-amino acid polypeptide which is mainly synthesized in adipose tissue, muscle, pancreas, and macrophages. Resistin is believed to have a role in insulin resistance and obesity [91]. As a proinflammatory cytokine, resistin is also believed to be related to inflammatory diseases independently from insulin resistance. The association between thyroid diseases and resistin levels is not well known. Ziora et al. described a positive relationship between serum resistin levels and FT4 in anorexia nervosa patients [92]. Iglesias et al. reported that serum resistin levels were similar in hypothyroid and euthyroid subjects [84]. Kaplan et al. demonstrated that no short-term significant changes in resistin, leptin, and adiponectin levels occurred in thyroidectomy-induced hypothyroidism when compared to the euthyroid state [93]. Another study showed a positive association between resistin levels and thyroid hormones [81]. Alterations in resistin levels by other adipocytokines can be the reason for this conflicting data on resistin levels and thyroid status. It is suggested that changes in resistin levels can act as an adaptive mechanism in thyroid dysfunction. 

## 8. Conclusion

There is complex interrelation between adipocytokines with thyroid gland and pituitary. Recent studies investigating the associations between thyroid functions and adipocytokines are conflicting due to different patient characteristics, coexisting autoimmunity, and probably nutritional status. New adipocytokines that will be discovered in near future also may have relation with thyroid gland diseases. Also balance between several adipocytokines that have role in thyroid metabolism and thyroid cancer may be more important. Combination of these adipocytokines may be more helpful. 
